# Platelets miRNA as a Prediction Marker of Thrombotic Episodes

**DOI:** 10.1155/2016/2872507

**Published:** 2016-11-30

**Authors:** Michal Bijak, Malgorzata Dzieciol, Joanna Rywaniak, Joanna Saluk, Marzenna Zielinska

**Affiliations:** ^1^Department of General Biochemistry, Faculty of Biology and Environmental Protection, University of Lodz, Pomorska 141/143, 90-236 Lodz, Poland; ^2^Intensive Cardiac Therapy Clinic, Medical University of Lodz, Pomorska 251, 91-213 Lodz, Poland

## Abstract

The blood platelets are crucial for the coagulation physiology to maintain haemostatic balance and are involved in various pathologies such as atherosclerosis and thrombosis. The studies of recent years have shown that anucleated platelets are able to succeed protein synthesis. Additionally, mRNA translation in blood platelets is regulated by miRNA molecules. Recent works postulate the possibility of using miRNAs as biomarkers of atherosclerosis and ischemic episodes. This review article describes clinical studies that presented blood platelets miRNAs expression profile changes in different thrombotic states, which suggest use of these molecules as predictive biomarkers.

## 1. Background

According to the World Health Organization statistics, cardiovascular disease causes nearly half of the deaths in developed countries. A worrying trend is the increase in the incidence of young people despite the growing awareness of healthy life style, the role of physical activity, and proper diet [1]. Myocardial infarction (MI) in persons under the age of 45 years accounts for 6% to 10% of this type of incidents. Unlike older patients, half of young patients have single-vessel coronary disease, and, in up to 20%, the cause is not related to atherosclerosis. One of the important risk factors for MI was family history of disease [2]. “CONFIRM” study conducted from 2003 to 2009 shows that positive family history of MI is the strongest clinical predictor of future myocardial infarction in young patients [3]. All of these evidences indicate the need for a detailed analysis of the genetic basis of the pathogenesis of thrombosis. It has been shown that miRNAs regulate the biological response of platelets: change of their shape and secretion of granules content [4]. miRNA profiling has been shown to be more accurate than mRNA expression profiling in characterizing the differentiation of multiple human cancers [5]. That postulates the possibility of using platelets miRNAs as predictive biomarkers of thrombotic events. This review article describes clinical studies that presented blood platelets miRNAs expression profile changes in different thrombotic states, which suggest use of these molecules as predictive biomarkers.

## 2. The Role of Platelets in Thrombotic Events

**Figure 1:**
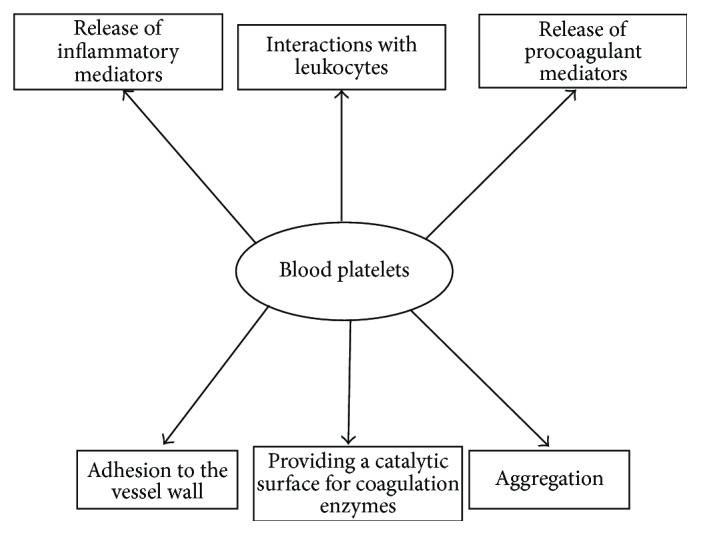
Scheme presented role of blood platelets in hemostasis.

The blood platelets are crucial for the coagulation physiology to maintain haemostatic balance and are involved in various pathologies such as atherosclerosis and thrombosis. Due to a large number of specific membrane receptors blood platelets are high reactive cells, readily activated by many physiological and nonphysiological agonists. The signaling pathways* via* specific receptors are dependent on the type of agonists but they always lead to physiological responses expressed as platelet activation [6]. The expression of multiple membrane receptors, both constitutive and activation-dependent, mediates platelet adhesion and aggregation at sites of vascular injury. In primary haemostasis activation of blood platelets leads to formation of platelet plug that seals the breach in the vessel wall and prevents excess blood loss [7, 8]. Subsequently, the activated platelets facilitate secondary haemostasis which supports the formation of a fibrin clot by carrying coagulation factors and providing a catalytic surface for the major interactions of the coagulation cascade [9–11] (Figure 1).

The platelet activation mediated by a complex series of intracellular processes involved in haemostasis, thrombosis, and inflammation is one of the most important risk factors in the cardiovascular system disturbance, associated with the occurrence of thromboembolic complications [12]. Thromboembolic complications leading to ischemic acute coronary syndromes, stroke, and deep vein thrombosis are the reason of death or chronic conditions that limit the quality of life and generate high costs of therapy and care.

The acute coronary syndrome (ACS) refers to group of clinical symptoms compatible with acute myocardial ischemia and includes unstable angina (UA), non-ST-segment elevation myocardial infarction (NSTEMI), and ST-segment elevation myocardial infarction (STEMI) [13]. It is well known that acute coronary syndromes with different clinical manifestations have a common pathophysiology, which is associated with coronary artery thrombosis [14]. The platelets are known to play a fundamental role in pathogenesis of ACS. Platelets are able to form pathogenic, occlusive intracoronary thrombus, leading to acute ischemic events [15]. The platelet adhesion and aggregate formation are critical events that occur in ACS. The patients with ACS exhibit increased reactivity and aggregation of blood platelets inside coronary circulation, which results in partial or complete obstruction of the coronary artery [16].

The platelets contribute to acute thrombosis with a multiple step mechanism: the first is adhesion of platelets to the endothelium. The interaction occurs between the constituents of the exposed subendothelium, including collagen, von Willebrand factor, fibronectin, and specific platelet surface membrane receptors. Thereby, platelets overcome the high blood shear forces and attach themselves to the target endothelium site. The binding of fibrinogen and selected matrix proteins containing Arg-Gly-Asp (RGD) sequences to integrin *α*IIb*β*3 (the most important and abundant platelet integrin) mediates stable platelet adhesion, aggregation, and thrombus formation. The further activation process occurs with a specific conformational change that induces the onset of multiple internal signaling networks. The hyperreactive platelets accelerate the formation of an intracoronary thrombus, leading to a cascade of clinical events [17]. The other distinctive feature of platelet activation is the release of the platelet microparticles (PMPs) by these cells. PMPs are the most plentiful cell microparticles found in the circulation [18–20] and as circulating sources of tissue factor (TF) that is a transmembrane protein involved in thrombin generation are potential mediators of blood coagulation. Moreover, a formation of platelet microparticles is associated with the exposure of phosphatidylserine at PMPs outer membrane surface. The mechanisms of interaction of PMPs with various cells may involve a membrane fusion, endocytosis, or interaction of microparticles with cell membrane receptors to stimulate cellular signaling events [20].

A recent study evaluated the association between hyperreactivity of platelets to adenosine diphosphate (ADP) and outcomes in patients with stable cardiovascular disease [21]. According to other researchers, the platelets not only play a role in the formation of coronary artery thrombosis, but also may be involved in the initiation and propagation of atherosclerosis, potentially through interaction of activated platelets with endothelial cells and leukocytes or through the release of various, stimulating inflammation mediators [22]. The resting or activated blood platelets, apart from the integrin receptors for adhesive proteins, possess on their surface the additional molecules responsible for interaction with other cells, such as ICAM-2 (intercellular adhesion molecule-2), JAM-A, JAM-C (junctional adhesion molecule) and PECAM-1 (platelet endothelial cell adhesion molecule-1), P-selectin, CD40/CD40L molecules, complement receptors, receptors for immunoglobulins (FcR), and Toll-like receptors [23].

The platelet activation leads to exocytosis of granule constituents and release of an arsenal of potent inflammatory and mitogenic substances into the local microenvironment, thereby altering chemotactic, adhesive, and proteolytic properties of endothelial cells. These platelet-induced modifications of the endothelial phenotype support chemotaxis, adhesion, and transmigration of monocytes to the site of inflammation. Platelets contain three types of specific secretory granules, such as dense granules, *α*-granules, and lysosomes [24], which after platelet activation release a variety of mediators: adhesion proteins (e.g., fibrinogen, fibronectin, vWF, thrombospondin, vitronectin, P-selectin, GPIIb-IIIa, receptor complex GPIba-V-IX, and collagen receptor GP VI), growth factors (e.g., PDGF, TGF-*β*, EGF, and bFGF), chemokines (RANTES, platelet factor 4 [PF4; CXC chemokine ligand 4 (CXCL4)], epithelial neutrophil-activating protein 78 [ENA-78; CXCL5]), cytokine-like factors (e.g., IL-1*β*, CD40L, and *β*-thromboglobulin), and coagulation factors (e.g., factors V, XI, XIII, PAI-1 [plasminogen activator inhibitor], *α*2-antiplasmin, TFPI [tissue factor pathway inhibitor], antithrombin, plasminogen, and protein S). These proteins act in a concerted and fine-regulated manner, influencing widely differing biologic functions such as cell adhesion, cell aggregation, chemotaxis, cell survival and proliferation, coagulation, and proteolysis. All of these molecules accelerate inflammatory processes and cell recruitment [25–28].

## 3. Protein Synthesis in Platelets

In the human body about 1 × 10^11^ platelets are made daily as a result of complex processes of differentiation, maturation, and fragmentation of megakaryocytes. Mature and fully differentiated megakaryocytes are equipped with all elements necessary for the production of platelets [29, 30]. It has been known for a long time that platelet proteins may have different origins: some are synthesized in megakaryocytes, and some derive directly from blood plasma [31]. However, the studies completed in recent years have shown that anucleated platelets are able to succeed protein synthesis. Both, platelet-specific granules and other organelles, such as numerous mitochondria and mRNA molecules, allow the synthesis of proteins in platelets [32]. The activated platelets produce many proteins which release and/or expression is not observed in resting cells. This observation has become a prerequisite for studies on the ability of platelets to protein synthesis [33]. Despite lack of nucleus, platelets have stable mRNA transcripts with a long life correlated with platelet lifespan. They contain a very small amount of the mRNA, which is approximately 2 × 10^−15^ g and this is about 12 500 times less than in nucleated cells [34]. The characteristics of platelets transcriptome by cDNA microarray analysis showed that the platelets contain thousands of base pairs coded information pieces derived from megakaryocytes. About 5000 mRNA transcripts in the blood platelets have been described so far, which represent a half of the amount of transcripts detected in megakaryocytes [35]. In 1989 Roth et al. [36] have discovered polyadenylated mRNA molecules in blood platelets. SAGE (Serial Analysis of Gene Expression) method also shows the presence of noncoding 3′-untranslated (3′-UTRs) regions in platelets mRNA, which are longer and more complicated in comparison to the same region in mRNA of other eukaryotic cells [37]. 3′-UTRs regions are located after coding sequence and play a role in posttranscriptional regulation. 3′-UTRs regions are signal for process called polyadenylation. In this process the enzyme called poly-A polymerase adds adenine nucleotides to the 3′ end of mRNA forming poly-A tail which is 100–250 adenine residues long. The presence of poly-A tail causes that mRNA molecules are more stable and prevents them against intracellular degradation. Additionally, the platelets' mRNA 3′-UTRs regions have also a large number of sequences rich in adenine and uracil ARE (ang. AU-rich elements) [33, 37] which regulate mRNA degradation by inhibition of shortening of poly-A tail [38]. According to Booyse and Rafelson Jr., the blood platelets' mRNA is more stable and resistant to degradation in comparison to the mRNA contained in other mammalian cells [39]. They demonstrated that blood platelets contain very stable mRNA transcripts with long lifespan correlated with platelet lifetime [40]. This is especially important in the case of anucleated platelets, which are not able to restore their mRNA pool.

## 4. The Role of miRNA in Platelet Protein Synthesis

**Figure 2:**
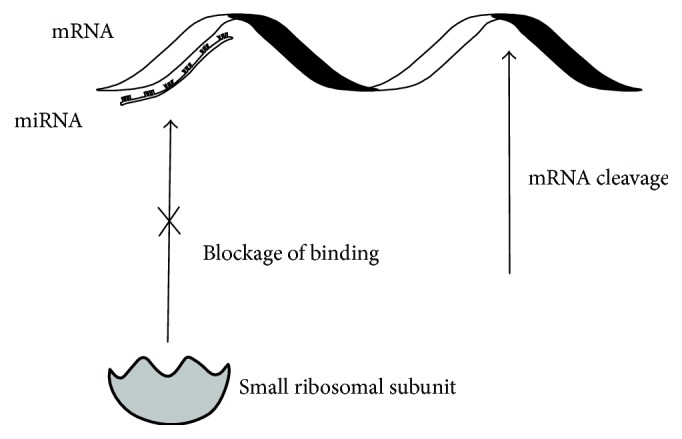
Role of miRNA in translation process.

The study carried out in 2009 by Landry et al. [41] shows that mRNA translation in blood platelets is regulated by miRNA molecules, which often hybridizes to the mRNA sequences localized in 3′-UTRs regions. MicroRNAs belong to class of small noncoding RNAs (21–24 nucleotides) and normally they negatively regulate the target mRNA expression at the posttranscriptional level. The miRNAs are generated from hairpin structures maturated by the RNAse III ribonucleases Drosha/Dicer to mature miRNAs. The mature miRNA molecules are incorporated into the complex containing Argonaute 2 protein (Ago-2). The mature miRNAs act as posttranscriptional regulators of gene expression by base pairing with mRNAs, thereby causing exonucleolytic mRNA decay or translational repression [42] (Figure 2). The mature miRNA together with the RNA-induced silencing complex (RISC) hybridizes to the mRNA sequence located 3′-UTR. The gene regulation by RISC complex is guided by sequence complementarity between the “seed region” (nucleotides 2–7 and 8) of the microRNA and the 3′-UTR of the mRNA. Recently, it has been also demonstrated that miRNAs may act as positive regulators in some cases. It is estimated that 1–4% genes in the human genome are miRNAs, and a single miRNA can regulate as many as 200 mRNAs. There is increasing evidence suggesting that miRNAs play critical roles in many key biological processes, such as cell growth, tissue differentiation, cell proliferation, embryonic development, and apoptosis. miRNAs play also important roles in cellular signaling network, cross-species gene expression variation and coregulation with transcription factors [43].

## 5. Platelets miRNA and Thrombotic Complications

The mutation of miRNAs, dysfunction of miRNA biogenesis, and dysregulation of miRNAs and their targets may result in various diseases. Currently, it has been reported that more than 70 diseases are associated with miRNAs (http://cmbi.bjmu.edu.cn/hmdd) [5]. Many studies have reported a great number of miRNA-disease associations and shown that the mechanisms of miRNAs involved in diseases are very complex. miRNA profiling has been shown to be more accurate than mRNA expression profiling in characterizing the differentiation of multiple human cancers.

Landry et al. [41] in 2009 first described 219 different types of miRNA in blood platelets expression profiles of which were different among platelets and megakaryocytes. Additionally, differential platelet miRNA profiles compared to neutrophils, were observed, what suggest the lack of leukocyte contribution to the platelet miRNA signals. The three most abundant miRNAs in blood platelets were miR-223, let-7c, and miR-19a. In support of this assertion platelets contain also pre-miRNA molecules and known protein components of the pre-miRNA processing complex, that is, RNase Dicer and TRBP2, as well as Ago-2, the core component of miRNA effector complexes. Moreover, RNase Dicer and TRBP2 form a complex which in platelets is catalytically active in pre-miRNA processing into miRNA like as megakaryocytes. It indicates the possibility of precursor miRNA maturation in the platelets. In contrast, the nuclear microprocessor components Drosha and DGCR8 were not detected in blood platelets, which is consistent with their enucleate nature. The same study [41] demonstrates that platelets have functional protein complexes of miRNA (miR-223) and Ago-2, and these complexes specifically regulate expression of the functionally important platelet purinergic P2Y12 adenosine diphosphate receptor. Furthermore, there is an evidence which suggests that miRNA (miR 28) can modulate expression of the c-mpl thrombopoietin (Tpo) platelet receptor [44].

In 2011, Osman and Fälker [45] have identified 281 transcripts, of which 228 were mature miRNA and 53 minor miRNA. Six of these miRNAs (miR-15 a, miR-339-3 p, miR-365, miR-495, miR-98, and miR-361-3 p) were up- or downregulated in activated human platelets. The changes in the levels of some miRNAs in platelets were associated with thrombin stimulation response.

Nagalla et al. [46] detected in platelets 284 miRNA transcripts, 74 of which showed various expression depending on the platelet reactivity. However, only the expression of 7 miRNA (miR-19b, miR-34b, miR-190, miR-320a, miR-320b, miR-320c, and miR-320d) showed a strong correlation with the degree of platelet response to adrenaline. The most abundant miRNA in platelets is miR-223 followed by miR-126. The miR-96, miR-200b, miR- 495, miR-107, and miR-223 are critically involved in platelet reactivity, aggregation, secretion, and adhesion [47].

In 2012 Plé et al. [54] have discovered more than 492 different mature miRNA transcripts in platelets. The in vitro study demonstrated that human blood platelets are able to uridylate miRNA molecules, which indicates the presence in platelets of the uridyltransferase enzyme TUT4. Additionally, in this study authors detected numerous miRNA isoforms (isomiRs) resulting from imprecise maturation caused by ribonucleases Drosha/Dicer. This study unveils the existence of very varied and multifaceted microRNA pathway in human platelets which suggest important role of miRNA in blood platelets functioning.

In study performed by Sondermeijer group [48] 214 miRNA molecules were identified, which have different expression levels in blood platelets obtained from patients with premature coronary artery disease in comparison to healthy donors of blood platelets. After biostatistics analysis six miRNAs (miR340^*∗*^, miR615-5p, miR545:9.1, miR451, miR454^*∗*^, and miR624^*∗*^) remained significantly and more than 1.5-fold upregulated whereas miR-12801 was remained significantly and more than 1.5-fold downregulated. Two independent cohort studies indicate that two miRNAs (miR624^*∗*^ and miR340^*∗*^) are significantly upregulated in patients with coronary artery disease (CAD) as compared to healthy controls. The authors recommend using these molecules as potential blood platelets diagnostic markers of coronary artery disease.

The changes in blood platelets miRNA expression profiles were also observed in patient after acute coronary syndrome. In blood platelets of patients with STEMI the most downregulated miRNAs were miR186-5p and miR185-5p, whereas miR127-3p and miR221-3p were upregulated in these cells. While in blood platelets from patients with NSTEMI the most downregulated miRNAs were miR20a-5p and miR942, the most upregulated miRNA molecules were miR483-5p and miR146a-5p [49].

Another circulation pathology, where platelet miRNAs show different expression profiles, is atrial fibrillation. This condition is notoriously associated with heart failure (HF) which possesses a very negative prognosis. MiRNAs have different expression profiles in platelets of patients with systolic HF compared to controls without cardiac disease. MiR-150 expression level was more than 3-fold lower in blood platelets obtained from patients with HF with atrial fibrillation [50].

The clinical evidences suggest that vesicle-associated membrane protein 8 (VAMP8)/endobrevin, a critical v-SNARE involved in platelet granule secretion, may be associated with clinical arterial thrombosis. The studies performed on five independent patient populations demonstrated an association between myocardial infarction and the rs1010 SNP in VAMP8 [55, 56]. Additionally, the blood platelet hyperreactivity was also correlated with increased levels of mRNA for VAMP8. The VAMP8 expression was found to be regulated by the platelet miRNA-miR-96. MicroRNA-96 can bind to the 3′-UTRs region of VAMP8 mRNA which was detected in platelets. Various levels of miR-96 were also presented in blood platelets with differing reactivity. The mean miR-96 level was found to be 2.6-fold higher in the hyporeactive subjects than in the hyperreactive subjects [57].

In physiological coagulation process the potent agonist of blood platelets is thrombin, a major enzyme generated in coagulation cascade [58]. The thrombin-activated platelets change their shape, secrete the contents of their granules, and finally aggregate [59]. The receptors belonging to the family of Protease-Activated Receptor (PAR) are responsible for blood platelets response to the thrombin. PAR are members of seven-transmembrane G protein-coupled receptor family. On the human platelets surface receptors PAR-1 and PAR-4 are present [60]. Edelstein et al. [61] demonstrated different expression level of miR-376c correlated with different PAR-4 reactivity.

The changes in platelet miRNAs expression were also observed in the rabbit atherosclerotic plaque model. The study showed that, in comparison to normal control animals, miR-126 and miR-223 levels in platelets of atherosclerotic plaque of rabbit were reduced. Moreover, the levels of these miRNAs were correlated with plaque morphology [62].

The changes in platelet miRNA expression were found also in patients with sickle cell disease (SCD) with state of hypercoagulability resulting prothrombotic predisposition. The forty differentially expressed miRNAs were identified in platelet of SCD patients with a risk of thromboembolic complications. From 24 downregulated miRNA molecules, 14 came from three miRNA families: miR-154, miR-329, and miR-376 which are localized in 14q32 region [51].

The blood platelet miRNAs can be also a risk factor and biomarkers for ischemic stroke. The miR-144 level in platelets is higher in diabetes mellitus type 2 (T2DM) patients with ischemic stroke. The platelet miR-223 expression decreases in this group in comparison to T2DM patients without thromboembolic complications [52]. In other study performed by Duan et al. [53] the expressions of platelet miR-223 and miR-146a in patients with diabetes mellitus and ischemic stroke were significantly lower than in healthy donors. Additionally, the expression level of these two miRNAs was correlated with blood platelet activation rates.

## 6. Conclusions

The genomics and proteomics and innovative research methods, based on the molecular analysis and closely related to bioinformatics, have become in recent years the basis of diagnostic tests designed to determine the predisposition of human to morbidity of several diseases. A recent works postulates the possibility of using miRNAs as biomarkers of atherosclerosis and ischemic episodes [4, 63] (Table 1). The miRNAs present in platelets may exert important regulatory functions in synthesis of proteins, which are involved in platelet activation pathways associated with platelet hyperactivity leading to thrombus formation. Many studies demonstrated different platelet miRNA expression profile patterns between patients with ischemic episodes and controls. The modern laboratory diagnostic is not limited to the identification of early stages of coronary obstruction but thanks to molecular methods is capable of detecting a predisposition to the illness. The detection of specific changes in platelet miRNA expression profiles associated with hyperactivity of platelets may have important implications in the prevention of embolic incidents.

## Figures and Tables

**Table 1:** Changes in blood platelets miRNA levels in thrombotic states.

MicroRNA level changes	Disease	Reference
↑ miR340^*∗*^	Premature coronary artery disease	[48]
↑ miR615-5p		
↑ miR545:9.1		
↑ miR451		
↑ miR454^*∗*^		
↑ miR624^*∗*^		
↓ miR-12801		
↑ miR340^*∗*^	Mature coronary artery disease	
↑ miR624^*∗*^		

↓ miR186-5p	Acute coronary syndrome (STEMI)	[49]
↓ miR185-5p		
↑ miR127-3p		
↑ miR221-3p		
↓ miR20a-5p	Acute coronary syndrome (NSTEMI)	
↓ miR942		
↑ miR483-5p		
↑ miR146a-5p		

↑ miR-150	Heart failure with atrial fibrillation	[50]

↓ miR-154	Sickle cell disease	[51]
↓ miR-329		
↓ miR-376		

↑ miR-144	Diabetes mellitus type 2 patients with ischemic stroke	[52, 53]
↓ miR-146a		
↓ miR-223	Diabetes mellitus type 2 patients without ischemic stroke	
